# Brain Activation Changes While Walking in Adults with and without Neurological Disease: Systematic Review and Meta-Analysis of Functional Near-Infrared Spectroscopy Studies

**DOI:** 10.3390/brainsci11030291

**Published:** 2021-02-26

**Authors:** Alka Bishnoi, Roee Holtzer, Manuel E. Hernandez

**Affiliations:** 1 Department of Kinesiology & Community Health, University of Illinois at Urbana-Champaign, Urbana, IL 61801, USA; abishn2@illinois.edu; 2 Ferkauf Graduate School of Psychology, Yeshiva University, Bronx, NY 10461, USA; roee.holtzer@yu.edu; 3 Department of Neurology, Albert Einstein College of Medicine, Bronx, NY 10461, USA

**Keywords:** neuroimaging, dual-task walking, neurological disorders

## Abstract

(1) Functional near-infrared spectroscopy (fNIRS) provides a useful tool for monitoring brain activation changes while walking in adults with neurological disorders. When combined with dual task walking paradigms, fNIRS allows for changes in brain activation to be monitored when individuals concurrently attend to multiple tasks. However, differences in dual task paradigms, baseline, and coverage of cortical areas, presents uncertainty in the interpretation of the overarching findings. (2) Methods: By conducting a systematic review of 35 studies and meta-analysis of 75 effect sizes from 17 studies on adults with or without neurological disorders, we show that the performance of obstacle walking, serial subtraction and letter generation tasks while walking result in significant increases in brain activation in the prefrontal cortex relative to standing or walking baselines. (3) Results: Overall, we find that letter generation tasks have the largest brain activation effect sizes relative to walking, and that significant differences between dual task and single task gait are seen in persons with multiple sclerosis and stroke. (4) Conclusions: Older adults with neurological disease generally showed increased brain activation suggesting use of more attentional resources during dual task walking, which could lead to increased fall risk and mobility impairments. PROSPERO ID: 235228.

## 1. Introduction

Daily living activities such as walking rely on multiple neuronal structures [1]. Walking involves complex interactions between different cortical areas of the brain, which regulate attention and executive function, to avoid obstacles and safely navigate complex environments [2,3]. This ability to process and navigate properly can become compromised with age or in adults with neurological or musculoskeletal disorders [4].

Brain activity or cortical activity can be measured by various neuroimaging methods such as functional magnetic resonance imaging (fMRI), magnetoencephalography (MEG), positron-emission tomography (PET), electroencephalography (EEG), and functional near-infrared spectroscopy (fNIRS). Although fMRI is considered gold standard for the assessment of activity in cortical areas, it suffers from the susceptibility of motion artifacts and restrictions in doing walking activities [5,6,7]. Similarly, MEG exhibits high vulnerability towards motion artifacts [6], while PET doesn’t allow repeated measurement due to injection of radioactive tracers [8]. On the other hand, EEG has weak spatial resolution, takes time to prepare, is vulnerable to artifacts and signal processing is difficult for nonexperts in the field [9,10,11]. Due to these restrictions among neuroimaging techniques, Functional Near Infrared Spectroscopy (fNIRS) has been used to record the cortical activity while walking. fNIRS is an optical neuroimaging technique for assessing cortical activity through the hemodynamic response of the brain while the participant walks freely [12], especially while dual task walking.

A dual task (DT) paradigm is a behavioral procedure in which individuals are required to perform two tasks simultaneously. Dual tasks can involve a wide range of concurrent sensory, motor, or cognitive tasks, and may be analogous to activities in daily life such as cooking, shopping, or walking while talking. Despite such variety, dual tasking often results in decline in performance in one component or both components of the dual task as compared to the single task, which is called dual task interference or dual task cost. The concept of capacity limitation in cognitive resource and performance limitation have been used to explain this dual task interference [13]. DT paradigms have received significant interest among researchers as it provides a gold standard for evaluation of the “central executive system” [14,15]. Moreover, in the context of gait, the use of DT paradigms allows researchers to assess the causal effect attentional resources have on walking performance under circumstances that better approximate real life conditions [16,17]. In comparison with a single task condition, DT conditions have shown activation in the dorsolateral prefrontal cortex of the brain [14,18]. This pattern of increased cortical activation in the prefrontal cortex has also been demonstrated through fNIRS studies of dual task walking.

There seems to be considerable amount of literature on fNIRS measuring cortical activity in areas such as the PFC, Pre-Motor Cortex (PMC), Supplementary Motor Area (SMA), and Sensory Motor Cortex (SMC) while performing dual tasks. However, previous fNIRS reviews have described the history of fNIRS [19], modelling and analysis of fNIRS [20,21], comparison of patterns of cortical activity using a variety of imaging techniques in walking studies [1,4], methodological approaches in postural and walking studies [22], data processing techniques [23], and PFC activations measured during walking [24] or during cognitive and motor tasks [25]. However, no review has undertaken a quantitative synthesis of brain activation differences in adults while dual task walking and how these effects differ among adults with and without neurological diseases.

Given the increased usage of fNIRS in recent years to study cortical control of locomotion, this study was designed to address an important gap in the literature: What are the brain activation differences while dual task walking in adults with neurological diseases? Specifically, we systematically reviewed and quantitatively synthesize brain activation differences, assessed using fNIRS, in adults with and without neurological disease while dual-task walking. The aims of this systematic review were to: (1) Quantify the changes in cortical activation patterns between different dual tasks; (2) quantify activation differences between different populations; (3) evaluate each study based on the quality assessment criteria.

## 2. Materials and Methods

### 2.1. Study Selection Criteria

Studies that met all of the following criteria were included in the review: (1) Study design: Cross-sectional, randomized controlled trial (RCT), cohort study, pre-post study; (2) population: Adults 18 years and older with or without neurological disease; (3) measured walking in the dual task; (4) used fNIRS to quantify oxygenated hemoglobin (HbO2) as the outcome measure; (5) was a peer-reviewed article; (6) was published in English; (7) Subject: Humans; and (8) Timespan: All years. Studies were excluded if: (1) fNIRS was not used; (2) dual task didn’t involve walking; (3) conference proceeding or review article; or (4) was a non-English publication.

### 2.2. Search Strategy

The systematic review and meta-analysis described in the Preferred Reporting Items for Systematic Reviews and Meta-analysis process [26] were adopted to guide the review process. A keyword search was performed in PubMEd, Cumulative Index of Nursing and Allied Health, PsycINFO, Scopus and Web of Science from August 2019–June 2020. The search algorithm included all possible combinations of keywords from the five groups: (1) “Dual”; (2) “task” or “motor skill”; (3) “gait”, “locomotion”, “walking”, “ambulation”; (4) “adults”; (5) “neuroimaging” or “fNIRS” or “functional near infra*”. The specific search algorithm for each database is provided in Appendix A.

Titles and abstracts of the articles identified through the keyword search were screened for the study selection criteria. Two reviewers (A.B. and M.E.H.) independently conducted title and abstract screening to determine their eligibility. Interrater agreement was determined by Intraclass correlation coefficient value and authors showed excellent correlation (ICC = 0.84). Any disagreements were resolved by discussion. A cited reference search (i.e., forward reference search) and reference list search (i.e., backward reference search) were conducted on the full text articles that met the study eligibility criteria from the keyword search. Articles identified through forward and backward search were further screened and evaluated by using the same study selection criteria. Reference searches were repeated on all newly identified articles until no additional relevant articles were found. 

### 2.3. Data Extraction

A standardized data extraction form was used to collect methodological and outcome variables from each selected study including author(s), year of publication, study design, sample size, participant characteristics (i.e., gender, age, pathology), single-task type, dual-task type, outcome measures (HbO2, Hb mean and SD) and key findings in terms of effect of dual-task on walking in adults assessed by fNIRS was extracted. 

### 2.4. Quantitative Data Synthesis

Meta-analysis was performed to estimate the pooled effect size for PFC activation, measured by the oxygenated hemoglobin (HbO2), furthermore, we performed subgroup analysis on fNIRS-derived HbO2 assessed during active walking under single and dual-task conditions. Secondary meta-analysis was done to find activation differences between single task and dual task in adults with and without neurological diseases among various studies (DT difference = DT mean-Single task mean). Several studies were excluded from meta-analysis because they didn’t have control group in the study or dual task wasn’t implemented in control group. Study heterogeneity was assessed using the I^2^ index. The level of heterogeneity represented by I^2^ was interpreted as modest (I^2^ ≤ 25%), moderate (25% < I^2^ ≤ 50%), substantial (50% < I^2^ ≤ 75%) or considerable (I^2^ > 75%) [27]. A fixed-model was estimated when modest to moderate heterogeneity was present, and a random-effect model was estimated when substantial to considerable heterogeneity was present [27]. Publication bias was assessed by a visual inspection of funnel plots and tested by Egger’s tests. All statistical analyses were conducted using the Stata 14.2 SE version (StataCorp, College Station, TX, USA). All analysis used two-sided *t*-tests and *p* values equal or less than 0.05 were considered statistically significant. 

### 2.5. Study Quality Assessment

We used the National Institutes of Health’s (NIH) Quality Assessment tool for Observational cohort and Cross-sectional studies to assess the quality of each included study [28] (https://www.nhlbi.nih.gov/health-topics/study-quality-assessment-tools, accessed on 21 October 2019). The following questions were used for the criteria: (1) Was the research question or objective of the study clearly stated? (2) Was the study population clearly specified and defined? (3) Were the inclusion and exclusion criteria for being the study prespecified and uniformly applied to all the participants? (4) Was sample size justification, power description or variance and effect estimated provided? (5) For analysis, were the exposure of interest measured prior to the outcomes being measured? (6) Were the exposure measures clearly defined and valid? (7) Were the outcome measures clearly defined and valid? (8) Were potential confounding variables measured and adjusted statistically? (9) Was dual task clearly defined and applied? (10) Was fNIRS applied to prefrontal cortex in the study? This assessment tool rates each study based on score range 0 (unmet), 1 (partially met), 2 (completely met). A study-specific global score ranging from 0 to 18 was calculated by summing up scores across all criteria. The study quality assessment helped measure the strength of scientific evidence but was not used to determine the inclusion of studies.

## 3. Results

### 3.1. Study Selection

**Figure 1:**
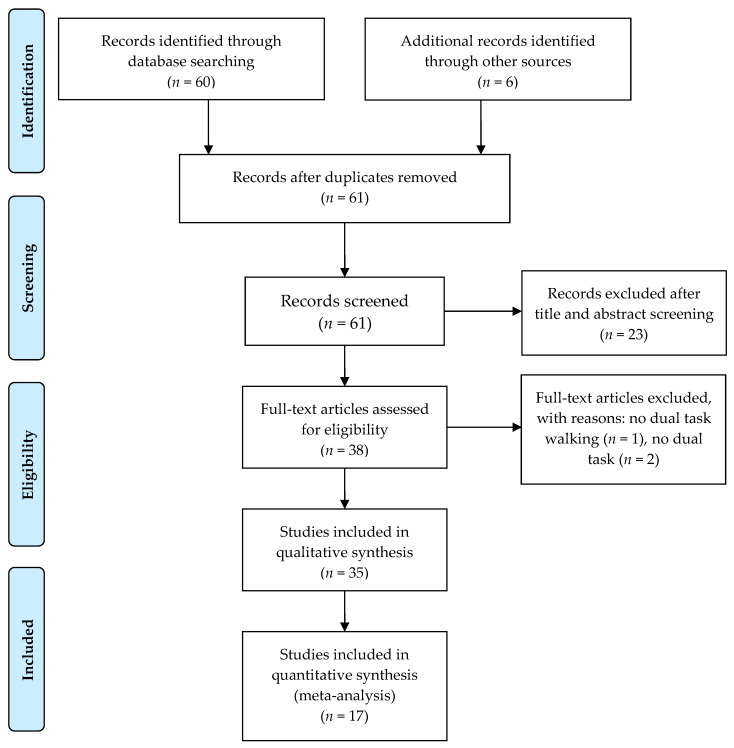
Systematic review PRISMA flow diagram.

As Figure 1 shows, a total of 61 articles were identified through keyword and reference search (forward/backward search), 23 of them were excluded in title and abstract screening. The remaining 38 articles were reviewed in full texts, and 3 of them is excluded for not meeting the study selection criteria as listed in Figure 1. The remaining 35 [29,30,31,32,33,34,35,36,37,38,39,40,41,42,43,44,45,46,47,48,49,50,51,52,53,54,55,56,57,58,59,60,61,62,63] articles were included in the review. 

### 3.2. Basic Characteristics of Included Studies

Table 1 and Table 2 reports the data extraction of 35 articles included in the review. As per basic characteristics of the studies, there are 10 studies including 178 healthy young adults aged between 19–39 years old; and 24 studies, including 2184 participants aged >55 years old. In neurological population, there are 4 studies including 175 people with Parkinson’s disease aged >60 years old; 5 studies including 100 people with Stroke aged >52 years old; and 3 studies, including 32 people with Multiple Sclerosis aged >50 years old. Lastly, there is only one study, including 16 people with Mild Cognitive Impairment aged >70 years old. 

Table 2 describes the oxyhemoglobin and deoxyhemoglobin outcomes of the studies while single task walking and dual task walking. There were 22 studies involving healthy young or older adults’ data while doing dual task walking [31,32,33,34,35,36,37,38,40,41,42,45,46,47,48,51,54,55,56,57,59,60]; out of which 17 involve letter generation tasks as a cognitive task while walking [31,32,33,34,35,36,37,42,46,51,55,56,57,60]; and 7 studies involve serial subtraction tasks while walking [38,39,45,47,48,54,59]; and 3 involved obstacle walking [38,47,57]. In addition, there were 4 studies showing PFC activation comparison between healthy older adults and people with Parkinson’s disease while doing serial subtraction as a cognitive task while walking [29,43,44,50]; and 2 studies did obstacle walking [43,44].

Appendix B provides further details on whether the study consisted of over-ground walking or treadmill walking, provided instruction for prioritization, or addressed systemic confounders. 

### 3.3. Meta-Analysis

For overall meta-analysis, we did find increase in PFC activation among single task and dual task walking conditions, relative to standing baselines prior to the task. For normal walking, total number of studies included in the random effect model are 24; overall effect size was significant (z = 6.89; *p* < 0.01), further, subgroup analysis showed that even normal walking can show significant increase in PFC activation among healthy older adults (z = 4.51; *p* < 0.01), people with stroke (z = 2.06; *p* < 0.05) and multiple sclerosis (z = 2.64; *p* = 0.008). 

**Figure 2:**
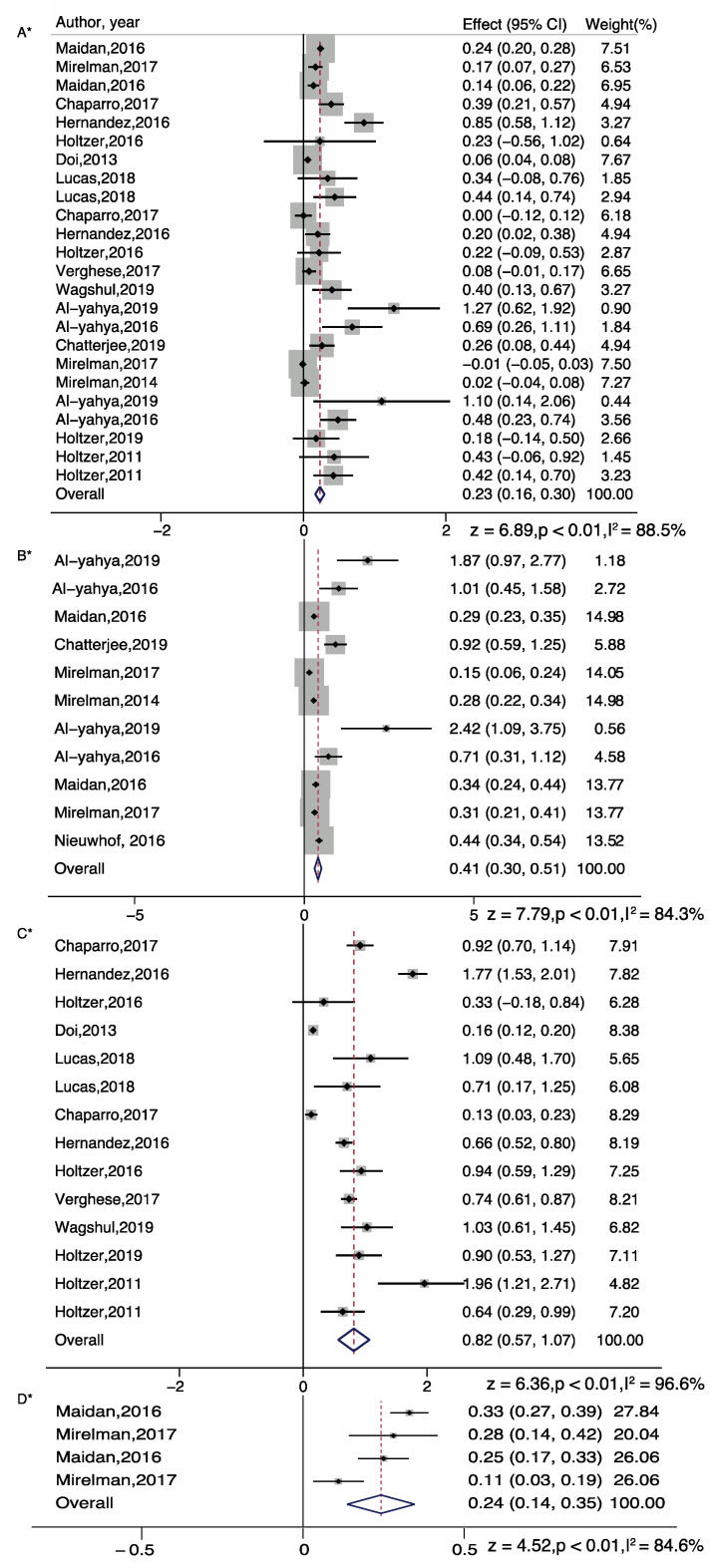
Brain activation differences while doing single task walking (**A**), serial subtraction task (**B**), letter generation task (**C**), or obstacle walking task (**D**). Note: * represents Random plot meta-analysis [29,30,31,35,36,37,42,44,47,48,50,52,55,56,58,62,63].

For dual task walking, we separated the meta-analysis for serial subtraction, obstacle walking and letter generation tasks. Overall, all tasks showed significant increase in PFC activation (Serial Subtraction, z = 7.79; *p* < 0.001; Obstacle walking, z = 4.52; *p* < 0.001; Letter generation, z = 6.36; *p* < 0.001). The effect of serial subtraction and letter generation task was highest among the three tasks (Figure 2).

**Figure 3:**
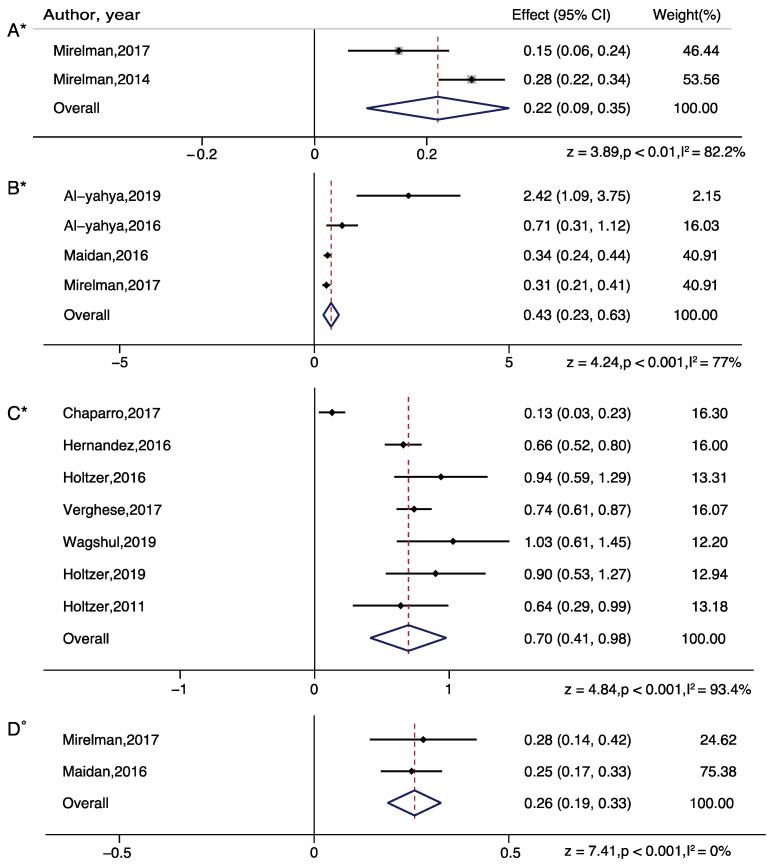
Brain activation differences while doing serial subtraction tasks in healthy young adults (**A**), and in serial subtraction tasks (**B**), letter generation tasks (**C**), or obstacle walking tasks (**D**) in healthy older adults. Note: ° represents Fixed plot meta-analysis, * represents Random plot meta-analysis [29,30,31,35,36,44,47,48,52,55,56,62].

**Figure 4:**
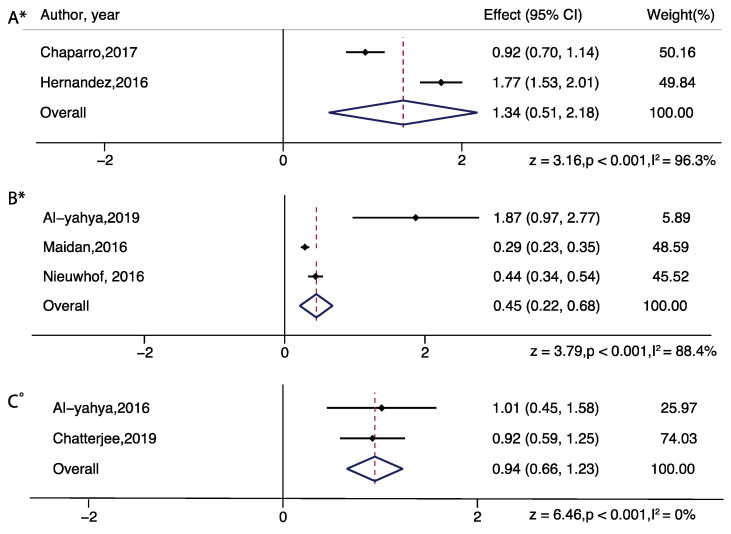
Brain activation differences while doing letter generation task (**A**) and serial subtraction task (**B**,**C**) in older adults with neurological diseases (multiple sclerosis-A, Parkinson’s disease-B, stroke-C). Note: ° represents Fixed plot meta-analysis, * represents Random plot meta-analysis [29,30,44,50,52,62,63].

For subgroup analysis in serial subtraction, a significant increased effect was found in all groups: healthy young adults (z = 3.89; *p* = 0.001), healthy older adults (z = 4.24; *p* < 0.001), people with Parkinson’s disease (z = 3.79; *p* < 0.01), people with stroke (z = 6.46; *p* < 0.001). For subgroup analysis in obstacle walking, a significant increased effect was found in healthy older adults only (z = 7.41; *p* < 0.001). For subgroup analysis in letter generation task, a significant increased effect was found in healthy older adults (z = 4.84; *p* < 0.001) and people with multiple sclerosis (z = 3.16; *p* = 0.002) (Figure 3 and Figure 4). 

**Figure 5:**
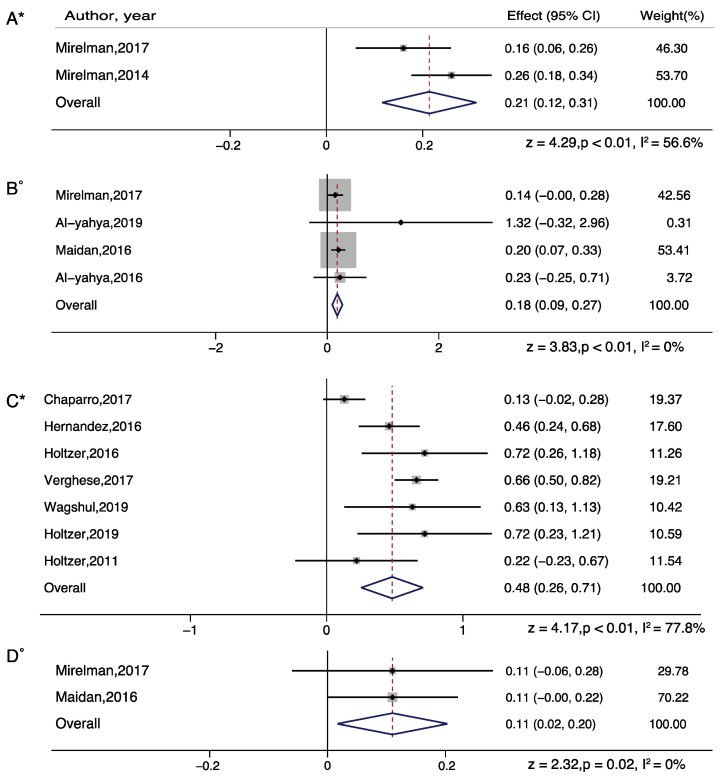
Dual task brain activation differences while doing serial subtraction tasks in healthy young adults (**A**), and in serial subtraction tasks (**B**), letter generation tasks (**C**), and obstacle walking tasks (**D**) in healthy older adults. Note: ° represents Fixed plot meta-analysis, * represents Random plot meta-analysis [29,30,31,35,36,44,47,48,52,62].

**Figure 6:**
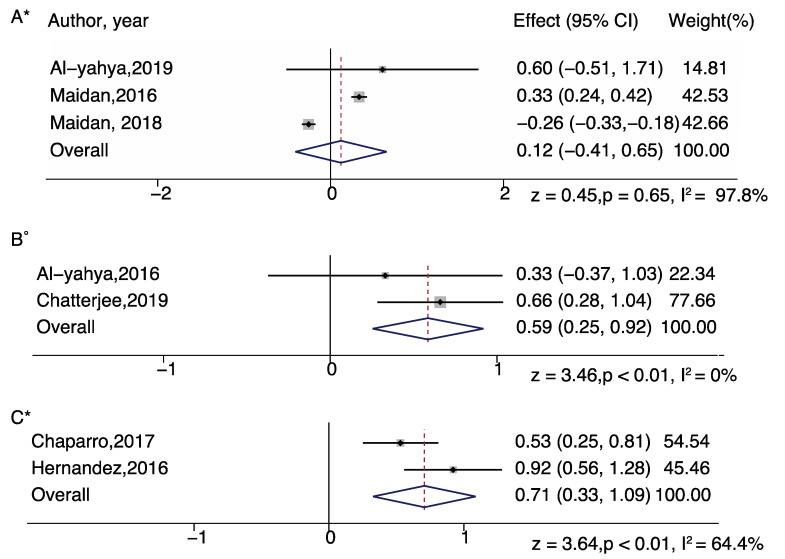
Dual task brain activation differences while doing serial subtraction (Parkinson’s disease—(**A**) and stroke—(**B**)), letter generation tasks (multiple sclerosis—(**C**)) in adults with neurological diseases. Note: ° represents Fixed plot meta-analysis, * represents Random plot meta-analysis [29,30,43,44,52,62,63].

In addition, we performed a DT difference meta-analysis (DT diff = DT mean- ST mean) which showed that letter generation tasks (z=4.17, p<0.01), serial subtraction (z = 3.83, *p* < 0.01), and obstacle walking (z = 2.32, *p* = 0.02) tasks demonstrated significantly increased PFC activation, in healthy older adults. In healthy young adults, serial subtraction tasks showed significant activation differences (z = 4.28, *p* < 0.01) and in adults with neurological diseases, letter generation tasks in persons with multiple sclerosis (z = 3.64, *p* < 0.01) and serial subtraction in persons with stroke (z = 3.46, *p* < 0.01) demonstrated significantly increased PFC activation, relative to single task walking (Figure 5 and Figure 6).

**Figure 7:**
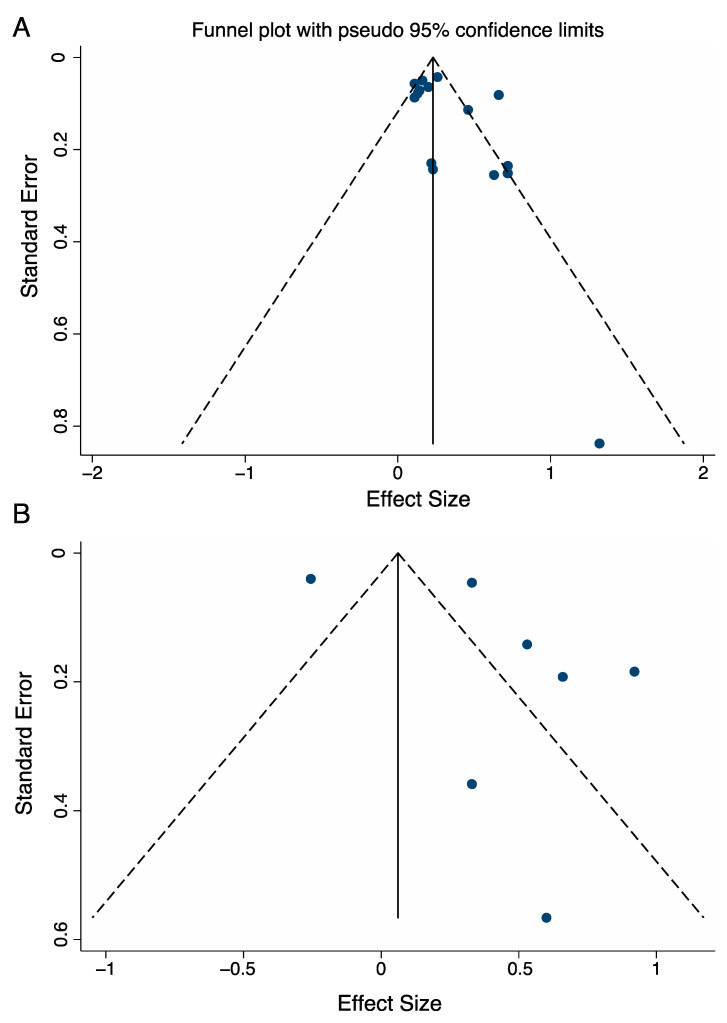
Funnel plots used to examine publication bias in dual task—single task differences in PFC activation in healthy adults (**A**) and in adults with neurological diseases (**B**).

Lastly, we did a publication bias analysis on the DT difference effect sizes. Egger’s test indicated no presence of publication bias in healthy adults across all reported dual tasks (*n* = 15, *p* = 0.095, Figure 7), nor in adults with neurological conditions across all tasks (*n* = 7, *p* = 0.233).

### 3.4. Study Quality Assessment

Table 3 reports results of our study quality assessment. Studies included in the review on average scored 16.26 out of 20 and ranged between 10 and 18. The distribution of qualification differed substantially across criteria. Thirty-four out of the 35 studies included in the review clearly described their study population except one study [45]. Six studies out of 35 failed to specify and apply inclusion and exclusion criteria to all participants [38,40,41,45,46,48]. Further, 12 studies didn’t provide sample size justification [30,38,40,41,43,44,45,49,51,52,54,61]. In term of analysis or paper, exposure of interest was not measured prior to outcome measure in most of the studies except one study [43], also one study did not define their outcome measures completely [30]. Last, the effect of cofounding variables was not measured and adjusted statistically in 12 studies [29,30,39,40,41,45,49,51,58,59,61,63]. 

## 4. Discussion

This study systematically reviewed and quantitatively synthesized existing scientific evidence on the differences in brain activation during walking while performing cognitive tasks among healthy young adults, healthy older adults, people with Parkinson’s disease, stroke and multiple sclerosis. PFC activation of adults with and without neurological disease participated in 35 studies was examined. This systematic review explicitly targeted: (1) quantifying the changes in cortical activation patterns between different dual tasks; (2) quantifying activation differences among different populations; (3) evaluating each study based on the quality assessment criteria. Overall, we show that the performance of obstacle walking, serial subtraction and letter generation tasks while walking result in significant increases in brain activation in the prefrontal cortex relative to standing or walking baselines in adults with and without neurological conditions. Consistent with previous work [24], our meta-analysis showed that letter generation tasks have the largest brain activation effect sizes relative to walking, and that significant differences between dual task and single task gait is seen in persons with stroke, using serial subtraction tasks, and in persons with multiple sclerosis while using letter generation tasks. Furthermore, we found that even normal walking can show significant increases in PFC activation among healthy older adults, people with stroke, and persons with multiple sclerosis. 

In terms of aging effects on dual task walking, results were found to differ depending on the baseline used. Larger effect sizes were observed in healthy older adults relative to healthy young adults in serial subtraction tasks while walking, relative to standing baselines (Figure 3), but larger effect sizes were observed in healthy young adults relative to older adults when using single task walking as a baseline (Figure 5). These findings suggest that normal walking may require additional attentional resources in healthy older adults, relative to young adults. These findings are consistent with studies examining age-related changes while dual task walking, suggesting no differences between healthy young and older adults while performing a visual check task [41], increases in older adults, relative to younger adults, while performing obstacle navigation [47], or decreases in older adults, relative to younger adults, while performing a letter generation task [35]. The discrepancy in results may arise from the effective lateralization in young adults, and utilization of additional cognitive resources to maintain gait performance in older adults, which is in accordance with the CRUNCH (Compensation-Related Utilization of Neural Circuits Hypothesis) model [64]. The CRUNCH model states that at low loads of cognitive demand, older adults recruit more cortical regions in comparison to young adults who demonstrate more focal activation based on the task. Thus, differences in concurrent task difficulty and the nature of the task may explain the discrepancy in age-related PFC activation changes while walking and could benefit from additional fNIRS studies with wider spatial coverage and varying difficulty levels to further our understanding of age-related changes.

Consistent with prior findings [24,36,58], we found that the performance of obstacle walking, serial subtraction and letter generation tasks while walking result in significant increases in brain activation in the prefrontal cortex relative to standing or walking baselines in adults with neurological conditions. Significant differences between dual task and single task gait are seen in persons with stroke while using serial subtraction tasks and in persons with multiple sclerosis while using letter generation tasks, even after significant increases in PFC activation while normal walking in both of these populations. The increases in PFC activation observed across adults with neurological conditions is consistent with increased inefficiency in PFC recruitment [65,66,67] or decreased automaticity of control in walking conditions in neurological populations [68]. 

Serial subtraction tasks while walking was found to have the largest effect sizes of PFC activation, relative to a standing baseline, consistent with observed brain activation increases of bilateral prefrontal areas in young healthy adults while performing serial subtraction tasks using functional magnetic resonance imaging [69]. Furthermore, relative to walking baselines, letter generation tasks, such walking while reciting alternate letters of the alphabet, were found to have the largest effect sizes of PFC activation, which is suggestive of compensatory cortical activation strategies in adults with neurological conditions, as reduced grey matter volumes in the PFC have been associated with greater increases in overall PFC activation from single task to dual task walking [56]. Thus, as different verbal tasks have distinct structural and functional brain correlates, and fluency tasks such as letter and category fluency have shared distinct neural correlates, small differences between tasks may allow for further clarification on the cortical structures most crucial for controlling gait in complex environments. Differences in PFC activation levels across the neurological populations also raises an important point of differences in cognitive function among these adults. Increases in PFC activation in a difficult working memory task such as serial subtraction task could be originating from prioritization of the task relevant areas as a consequence of further limited resources present in the brain [70]. Furthermore, other areas of cortical activation may be recruited for the support of dual task walking, depending on the cognitive task involved while walking and the specific neurological condition. Thus, care should be taken to utilize specific dual task walking paradigms whose activation can be captured by a given region of interest with fNIRS. 

### 4.1. Clinical Implications

Dual task walking paradigms may better approximate locomotion demands in natural settings where individuals are required to negotiate visual and auditory stimuli that interfere with the maintenance of safe gait. The ecological validity of such paradigms may thus confer improved predictive utility. For example, in older adults, worse dual task walking performance was associated with increased risk of incident frailty, disability and mortality even when adjusting for single task walking [71]. Poor dual-task walking is also predictive of falls in aging and neurological populations [72]. Specifically, with respect to fNIRS-derived HbO_2_ in the PFC, as assessed during walking, higher activation during dual but not single task conditions predicted increased risk of incident falls [55]. This finding suggested that over activation of the PFC during cognitively demanding walking was indicative of inefficient utilization of brain resources that predisposed individuals to greater falls risk. Establishing robust and reliable effect sizes for fNIRS-derived HbO_2_ during active walking in normal and disease populations is critical to determine its potential clinical utility as a tool for risk assessment of mobility-related decline and disability outcomes. Furthermore, within session training in dual-task walking resulted in improved performance and reduced fNIRS-derived HbO_2_ in the PFC (i.e., improved neural efficiency) in older adults [31]. This improvement in PFC efficiency was due to within session training in dual-task walking, however, was moderated by the presence of mild cognitive impairments [73] and fear of falling [32], an important risk factor for falls and other mobility-related outcomes [74,75,76,77,78]. The utility of fNIRS-derived HbO_2_, assessed during dual-task walking, as primary or secondary outcomes in clinical trials will have to be established in future research. fNIRS-derived measures may help ensure that dosage of any exercise prescription is comparable across individuals [79]. One limitation of current research is concerned with the variety of dual-task paradigms and experimental procedures used in different studies, as seen in other reviews [80]. While the robust effects sizes reported in the present study are encouraging standardization of tasks and procedures would be a necessary step moving forward. It is noteworthy that such efforts exist not only in traditional self-report, behavioral and cognitive outcomes in clinical research but also in recent neuroimaging outcomes [24]. A recent study revealed that using different filters and processing algorithms yielded some differences in the extracted values of HbO_2_ and Hb values of walking under single and dual talking conditions though the task effect remained similar [81]. Establishing transparent consensus criteria for fNIRS data processing, specifically with respect to dual task walking paradigms, will also be necessary for efforts to determine and enhance its utility in clinical research, as demonstrated by recent attempts to establish best practices [82,83].

### 4.2. Other Implications of fNIRS

Besides use of fNIRS in dual task paradigms, it is also used in community navigation tasks with augmented reality [84] or during environmentally complex tasks [85] in healthy young adults. This is due to the ability of fNIRS to work as a practical mobile neuroimaging device in complex real-world environments.

Measuring brain activity during natural environmental settings is a growing field and use of fNIRS has served as an advantageous tool, especially, in the area of Neuroergonomics, in which one can implement natural work settings and measure the brain activity of the individual while wearing fNIRS systems [86,87]. Lastly, while using fNIRS, researchers need to be aware of its application in different populations and in different experimental paradigms. Although, the focus of this systematic review was dual task paradigms, fNIRS has a wide range of potential applications.

### 4.3. Limitations

We found changes in the PFC activation among adults with and without neurological diseases, however, few studies have reported both HbO2 and Hb values, and the number of studies in each subgroup meta-analysis varied which may have led to biased findings. Although no publication bias was detected using Egger’s test in clinical populations nor in healthy adults, the small number of studies limits the power of the test to detect bias. The results of these studies should be interpreted with caution, as funnel plots suggest the presence of publication bias for the serial subtraction and letter generation dual tasks in patient populations but not in healthy older adults. We need more studies with these tasks to confirm our subgroup analysis. Given the limited number of studies, we were unable to carry out sub-group analyses on influencing factors such as overground versus treadmill walking, dual task prioritization, and processing of systemic confounders on PFC activation. Future work should examine the effect of these factors on PFC activation among adults with and without neurological diseases. 

## 5. Conclusions

The current review and meta-analytic study provide comprehensive information on PFC activation differences measured by fNIRS and yields novel information on the significance of cognitive tasks to be used while walking in adults with different neurological conditions. fNIRS technology seems to be a promising tool to shed light on the functioning of cortical areas in motor control. The information provided regarding the robustness of fNIRS-derived HbO_2_ assessed during active walking under single and dual-task conditions is a critical step towards establishing its utility as a risk factor of adverse cognitive and mobility outcomes in normal and disease populations. This information is also critical for establishing the potential utility of fNIRS-derived HbO_2_ as a treatment outcome measure that may enhance the monitoring and evaluation of the effectiveness of intervention programs among healthy adults and those with different neurological and non-neurological conditions. 

## Figures and Tables

**Table 1:** Study and Participant characteristics.

Study	Study ID	Year	Type of Study	Age (Mean ± SD (years))		Population	
				HYA	HOA	HYA	HOA
Beurskens et al. [41]	1	2014	Cross-sectional	25 ± 3	71 ± 4	15	10
Chen et al. [57]	2	2017	Cross-sectional	NA	78.1 ± 6	NA	90
Fraser et al. [59]	3	2016	Cross-sectional	22 ± 2	67 ± 5	19	14
George et al. [60]	4	2019	Cross-sectional	NA	76 ± 7	NA	325
Holtzer et al. ^†^ [35]	5	2011	Cross-sectional	19–29	69–88	11	11
Holtzer et al. ^†^ [36]	6	2016	Cross-sectional	NA	74 ± 6	NA	167
Holtzer et al. [37]	7	2016	Cross-sectional	NA	77 ± 7	NA	314
Holtzer et al. [34]	8	2017	Cross-sectional	NA	77 ± 7	NA	318
Holtzer et al. [33]	9	2018	Cross-sectional	NA	77 ± 7	NA	315
Holtzer et al. [31]	10	2019	Cross-sectional	NA	78 ± 6	NA	75
Holtzer et al. ^†^ [32]	11	2019	Cross-sectional	NA	78 ± 6	NA	83
Lin et al. [38]	12	2016	Cross-sectional	20–27	NA	24	NA
Lu et al. ^†^ [40]	13	2015	Cross-sectional	23 ± 2	NA	17	NA
Lucas et al. ^†^ [42]	14	2018	Cross-sectional	NA	75 ± 5	NA	55
Meester et al. [45]	15	2014	Cross-sectional	28 ± 6	NA	17	NA
Metzger et al. [46]	16	2017	Cross-sectional	28,19–39	NA	12	NA
Mirelman et al. ^†^ [48]	17	2014	Cross-sectional	31 ± 4	NA	23	NA
Mirelman et al. ^†^ [47]	18	2017	Cross-sectional	31 ± 4	70 ± 6	23	20
Osofundiya et al. [51]	19	2016	Cross-sectional	NA	81 ± 7	NA	20
Stuart et al. [54]	20	2019	Cross-sectional	20 ± 1	73 ± 8	17	18
Verghese et al. [55]	21	2017	Cross-sectional	NA	75 ± 6	NA	166
Wagshul et al. [56]	22	2019	Cross-sectional	NA	>65	NA	55
				**PD**	**HOA**	**PD**	**HOA**
Al-yahya et al. ^†^ [29]	23	2019	Cross-sectional	66 ± 6	60 ± 7	29	22
Maidan et al. ^†^ [44]	24	2016	Cross-sectional	72 ± 1	70 ± 1	68	38
Maidan et al. ^†^ [43]	25	2018	RCT	72 ± 1	NA	64	NA
Nieuwhof et al. [50]	26	2016	Cross-sectional	71 ± 5	NA	14	NA
				**Stroke**	**HOA**	**Stroke**	**HOA**
Al-yahya et al. ^†^ [30]	27	2016	Cross-sectional	60 ± 15	54 ± 9	19	20
Chatterjee et al. ^†^ [63]	28	2019	Cross-sectional	60 ± 10	NA	33	NA
Hermand et al. [61]	29	2019	Cross-sectional	71 ± 10	NA	11	NA
Liu et al. [39]	30	2018	Cross-sectional	52 ± 11	NA	23	NA
Mori et al. [49]	31	2018	Cross-sectional	61 ± 9	66 ± 1	14	14
				**MS**	**HOA**	**MS**	**HOA**
Chaparro et al. ^†^ [52]	32	2017	Cross-sectional	56 ± 5	63 ± 4	10	12
Hernandez et al. ^†^ [62]	33	2016	Cross-sectional	57 ± 5	61 ± 4	8	8
Saleh et al. [53]	34	2018	Cross-sectional	50 ± 8	50 ± 9	14	14
				**MCI**	**HOA**	**MCI**	**HOA**
Doi et al. ^†^ [58]	35	2013	Cross-sectional	75 ± 7	NA	16	NA

Note: Values are in mean ± SD or as otherwise indicated, ^†^ represents studies included in the meta-analysis. Abbreviations: MCI, Mild cognitive impairment; MS, Multiple Sclerosis; PD, Parkinson’s Disease; HOA, Healthy Older adults; HYA, Healthy Young Adults; RCT, Randomized Controlled Trial; NA, not available.

**Table 2:** Oxyhemoglobin (HbO_2_) & Deoxyhemoglobin (Hb) outcome measures for studies included in the review.

Study ID	Single Task	Mean ± SD			Dual Task	Mean ± SD		
		HYA		HOA		HYA		HOA
01		−0.13 ± 0.02		−0.09 ± 0.04	Walk & visual check	−0.15 ± 0.02		−0.23 ± 0.05
					WWT	−0.22 ± 0.02		−0.09 ± 0.03
02		NA		0.30 ± 1.21	WWT	NA		1.08 ± 1.51
					OW			
03		NA		NA	n-back task	1-back: 15.87 ± 5.62 ^◆^		1-back: 15.87 ± 5.62 ^◆^
						2-back: 13.67 ± 9.39 ^◆^		2-back: 13.67 ± 9.39 ^◆^
04		NA		0.11 ± 1.2 ^◆^	RAL	NA		0.705 ± 1.28 ^◆^
05 ^†^		0.43 ± 0.83		0.42 ± 0.49	RAL	1.96 ± 1.27		0.64 ± 0.60
06 ^†^		NA		0.22 ± 2.02	RAL	NA		0.94 ± 2.28
07		NA		0.11 ± 1.25	RAL	NA		0.73 ± 1.41
08		NA		0.11 ± 0.65	RAL	NA		0.66 ± 0.86
09		NA		0.11 ± 0.64	RAL	NA		0.7 ± 0.88
10		NA		0.215 ± 0.17	RAL	NA		0.995 ± 0.23
11 ^†^		NA		0.18 ± 1.51	RAL	NA		0.90 ± 1.72
12		Wide	−0.06 ± 0.26	NA	n-back walking Wide path Narrow path Obstacle path	1-back −0.92 ± 0.33 −0.47 ± 0.35 −1.05 ± 0.39	3-back −0.75 ± 0.31 −0.52 ± 0.23 −0.68 ± 0.27	NA
		Narrow	0.33 ± 0.36					
		Obstacle	−0.24 ± 0.36					
13 ^†^		NR		NA	SS7s	NR		NA
14 ^†^		NA		0.39 ± 0.97	RAL	NA		0.9 ± 1.54
15		0.22 ± 0.11 −0.1 ± 0.25 ″		NA	SS7s	0.36 ± 0.1 −0.15 ± 0.30 ″		NA
16		NA		NA	Letter generation task	NA		NA
17 ^†^		0.02 ± 0.03 ^◆^		NA	SS7s	0.28 ± 0.03 ^◆^		NA
					Counting back	0.18 ± 0.03 ^◆^		
18 ^†^		−0.01 ± 0.04 ^◆^		0.17±0.05 ^◆^	SS7s	0.15 ± 0.04 ^◆^		0.31 ± 0.05 ^◆^
					OW	0.11 ± 0.04 ^◆^		0.28 ± 0.07 ^◆^
19		NA		0.36 ± 0.40	Recite alternate letters	NA		1.145 ± 0.5
					Precision walking			1.595 ± 0.445
20		NA		NA	Digit vigilance task	−0.001 ± 0.07		−0.011 ± 0.07
21		NA		0.08 ± 0.62	RAL	NA		0.74 ± 0.85
22		NA		0.4 ± 1.04	RAL	NA		1.03 ± 1.58
		**PD**		**HOA**		**PD**		**HOA**
23 ^†^		1.27 ± 0.33 ^◆^ −0.76 ± 0.24 ^◆^″		1.10 ± 0.49 ^◆^ −0.82 ± 0.36 ^◆^″	SS7s	1.87 ± 0.46 ^◆^ −0.98 ± 0.34 ^◆^″		2.42 ± 0.68 ^◆^ −1.50 ± 0.50 ^◆^″
24 ^†^		0.24 ± 0.02 ^◆^		0.14 ± 0.04 ^◆^	SS3s OW	0.33 ± 0.03 ^◆^		0.25 ± 0.04 ^◆^
25 ^†^		−0.04 ± 0.035 ^◆^ −0.04 ± 0.035 ^◆^		NA	SS3s OW	−0.015 ± 0.035 ^◆^ 0.005 ± 0.035 ^◆^		NA
26		NA		NA	Counting forward SS Reciting digits	0.3 ± 0.07; 0.00 ± 0.05 ″ 0.44 ± 0.20; −0.02 ± 0.04 ″ 0.38 ± 0.15; −0.1 ± 0.19 ″		NA
		**Stroke**		**HOA**		**Stroke**		**HOA**
27 ^†^		0.69 ± 0.22 ^◆^ −0.45 ± 1.0 ^◆^″		0.49 ± 0.13 ^◆^ −0.4 ± 1.0 ^◆^″	SS7s	1.02 ± 0.28 ^◆^ −0.65 ± 1.0 ^◆^″		0.72 ± 0.21 ^◆^ −0.51 ± 0.99 ^◆^″
28 ^†^		0.26 ± 0.09 ^◆^ −0.1 ± 0.05 ^◆^″		NA	SS7s	0.92 ± 0.17 ^◆^ −0.2 ± 0.1 ^◆^″		NA
29		ST low	1.19 ± 0.7	NA	N-back	DT low	2 ± 2.24	NA
		ST high	1.23 ± 1.3			DT high	2.69 ± 2.22	
		walk	2.42 ± 1.93					
30		−5.3 ± 1.7 ^◆^^:^		NA	SS3s	18.67 ± 2.1 ^◆^^:^		NA
					WMT			
31 ^∆^		−0.3 ± 1.73		0.69 ± 2.11	SS3s	−0.073 ± 0.41		2.08 ± 1.87
		**MS**		**HOA**		**MS**		**HOA**
32 ^†^		0.39 ± 0.1 ^◆^		0 ± 0.06 ^◆^	WWT	0.92 ± 0.1 ^◆^		0.13 ± 0.06 ^◆^
33 ^†^		0.85 ± 0.14 ^◆^		0.2 ± 0.09 ^◆^	RAL	1.77 ± 0.12 ^◆^		0.66 ± 0.07 ^◆^
34		2.22 ± 0.91 ^◆^^:^		0.16 ± 0.95 ^◆^^:^	SS7s	1.64 ± 0.95 ^◆^^:^		3.18 ± 1.54 ^◆^^:^
		**MCI**		**HOA**		**MCI**		**HOA**
35 ^†^		0.06 ± 0.01 ^◆^		NA	Letter fluency task	0.16±0.02 ^◆^		NA

Note: ^◆^ represents standard error; ^†^ represents studies included in the meta-analysis; ^∆^ represents the different unit au.3; ^:^ represents the Hbdiff data (HbO_2_-Hb); ″ represents Hb data only. Abbreviations: WMT: walking motor task; NA: not available; NR: not reported; NW: normal walking; SS: serial subtraction; RAL: reciting alternate letters; WWT: walk while talk; OW: obstacle walking; MCI: mild cognitive impairment; MS: multiple sclerosis; PD: Parkinson’s Disease; HOA: Healthy older adults; HYA: Healthy young adults.

**Table 3:** Study Quality Assessment.

No.	Questions	Score
1	Was the research question or objective of the study clearly stated?	2
2	Was the study population clearly specified and defined?	1.94
3	Were inclusion and exclusion criteria for being in the study prespecified and uniformly applied to all participants?	1.66
4	Was sample size justification, power description or variance and effect estimates provided?	1.32
5	For analysis of paper, were the exposure(s) of interest measured prior to the outcome(s) being measured?	0.06
6	Were the exposure measures (independent variables) clearly defined, valid, reliable, and implemented consistently across all study participants?	2
7	Were the outcome measures (dependent variables) clearly defined, valid, reliable, and implemented consistently across all study participants?	1.97
8	Were potential confounding variables measured and adjusted statistically for their impact on the relationship between exposure(s) and outcome(s)?	1.32
9	Was dual task clearly defined and uniformly applied to all participants?	2
10	Was functional near infrared spectroscopy applied to prefrontal cortex part of brain and clearly defined in text?	2
	Total	16.26
	SD	0.59

## Data Availability

No new data were created or analyzed in this study. Data sharing is not applicable to this article.

## Appendix A

**Search Terms Used for Each Database:**	
**Database**	**Search Terms**
PubMed	((((((“dual”) AND “task” OR “motor skill” [Mesh] OR “motor skill”))) AND ((“gait” [Mesh] OR “gait” OR “locomotion” [Mesh] OR “locomotion” OR “walking” [Mesh] OR “walking” OR “ambulation” [Mesh] OR “ambulation”))) AND (“adults”)) AND ((“neuroimaging” [Mesh] OR “neuroimaging” OR “fNIRS” OR “functional near infra *”)) Species—Humans, Language—English.
Cumulative Index of Nursing and Allied Health	((“dual task”) AND (“walking” OR “gait” OR “locomotion” OR “ambulation”) AND (“adults”) AND (“neuroimaging” OR “fNIRS” OR “functional near infra *”)))Limiters: English language; Human, Journal article
Web of Science	((TS = ((“dual task”) AND (“walking” OR “gait” OR “locomotion” OR “ambulation”) AND (“adults”) AND (“neuroimaging” OR “fNIRS” OR “functional near infra *”)))) AND LANGUAGE: (English) AND DOCUMENT TYPES: (Article)Timespan: All years. Indexes: SCI-EXPANDED.
PsycINFO	((((((“dual”) AND “task” OR “motor skill” [Mesh] OR “motor skill”))) AND ((“gait” [Mesh] OR “gait” OR “locomotion” [Mesh] OR “locomotion” OR “walking” [Mesh] OR “walking” OR “ambulation” [Mesh] OR “ambulation”))) AND (“adults”)) AND ((“neuroimaging” [Mesh] OR “neuroimaging” OR “fNIRS” OR “functional near infra *”))Age: 18 yr & older; Language: English; Record type: Journal article; Population: Humans.
Scopus	( ( ( ( ( ( “dual” ) AND “task” OR “motor skill” [mesh] OR “motor skill” ) ) ) AND ( ( “gait” [mesh] OR “gait” OR “locomotion” [mesh] OR “locomotion” OR “walking” [mesh] OR “walking” OR “ambulation” [mesh] OR “ambulation” ) ) ) AND ( “adults” ) ) AND ( ( “neuroimaging” [mesh] OR “neuroimaging” OR “fNIRS” OR “functional near infra *” ) ) AND ( LIMIT-TO ( DOCTYPE, “ar” ) ) AND ( LIMIT-TO ( LANGUAGE, “English” ) ) AND ( LIMIT-TO ( SRCTYPE, “j” ) )Document type: Journal article; Language: English
Note: * represents wild card.	

## Appendix B

**Study ID.**	**Author**	**Treadmill or Overground Walking?**	**Prioritization of Dual Task?**	**Additional Noise Processing?**
1	Beurskens 2014 [41]	Treadmill	No	Yes
2	Chen 2017 [57]	Overground	Pay equal attention	Yes
3	Fraser 2016 [59]	Treadmill	Pay equal attention	NR
4	George 2019 [60]	Overground	Pay equal attention	NR
5	Holtzer 2011 [35]	Overground	Pay equal attention	Yes
6	Holtzer 2016 [36]	Overground	Pay equal attention	Yes
7	Holtzer 2016 [37]	Overground	Pay equal attention	Yes
8	Holtzer 2017 [34]	Overground	Pay equal attention	Yes
9	Holtzer 2018 [33]	Overground	Pay equal attention	Yes
10	Holtzer 2019 [31]	Overground	Pay equal attention	Yes
11	Holtzer 2019 [32]	Overground	Pay equal attention	Yes
12	Lin 2016 [38]	Overground	No	Yes
13	Lu 2015 [40]	Overground	Yes	Yes
14	Lucas 2019 [42]	Overground	Pay equal attention	Yes
15	Meester 2014 [45]	Treadmill	No	NR
16	Metzger 2017 [46]	Treadmill	No	NR
17	Mirelman 2014 [48]	Overground	No	Yes
18	Mirelman 2017 [47]	Overground	No	Yes
19	Osofyundiya 2016 [51]	Overground	Pay equal attention	NR
20	Stuart 2018 [54]	Treadmill	No	Yes
21	Verghese 2016 [55]	Overground	Pay equal attention	Yes
22	Wagshul 2019 [56]	Overground	Pay equal attention	Yes
23	Al-Yahya 2019 [29]	Treadmill	No	Yes
24	Maidan 2016 [44]	Overground	No	Yes
25	Maidan 2018 [43]	Treadmill	No	Yes
26	Nieuwhof 2016 [50]	Overground	NR	NR
27	Al-Yahya 2016 [30]	Treadmill	No	Yes
28	Chatterjee 2019 [63]	Overground	No	NR
29	Hermand 2019 [61]	Overground	Pay equal attention	Yes
30	Liu 2018 [39]	Overground	No	Yes
31	Mori 2017 [49]	Overground	No	NR
32	Chaparro 2017 [52]	Treadmill	No	Yes
33	Hernandez 2016 [62]	Overground	No	Yes
34	Saleh 2018 [53]	Overground	Pay equal attention	Yes
35	Doi 2013 [58]	Overground	No	NR
Note: NR = Not reported.				
